# Sugar-Sweetened Beverage Intake Among Adults, by Residence in Metropolitan and Nonmetropolitan Counties in 12 States and the District of Columbia, 2017

**DOI:** 10.5888/pcd17.190108

**Published:** 2020-01-23

**Authors:** Omoye Imoisili, Sohyun Park, Elizabeth A. Lundeen, Liping Pan, Terry O’Toole, Karen R. Siegel, Heidi M. Blanck

**Affiliations:** 1 Epidemic Intelligence Service, Center for Surveillance, Epidemiology, and Laboratory Services, Centers for Disease Control and Prevention, Atlanta, Georgia; 2 Division of Nutrition, Physical Activity, and Obesity, National Center for Chronic Disease Prevention and Health Promotion, Centers for Disease Control and Prevention, Atlanta, Georgia; 3 Division of Diabetes Translation, National Center for Chronic Disease Prevention and Health Promotion, Centers for Disease Control and Prevention, Atlanta, Georgia

## Abstract

The objective of this study was to describe the prevalence of sugar-sweetened beverage (SSB) intake among US adults (n = 68,896) residing in metropolitan and nonmetropolitan counties, by state, using data from the Behavioral Risk Factor Surveillance System. We used multinomial logistic regression to calculate adjusted prevalence ratios for daily (≥1 time per day) SSB intake. Overall, 26.0% of respondents reported daily SSB intake, with significantly higher prevalence in nonmetropolitan counties (30.9%) than in metropolitan counties (24.8%) (adjusted prevalence ratio = 1.32, 95% confidence interval, 1.26–1.39). This same pattern was significant in 5 of 11 states with metropolitan and nonmetropolitan counties. These findings could inform efforts to reduce frequent SSB intake in nonmetropolitan areas.

**Summary**

**What is already known about this topic?**

Frequent intake of sugar-sweetened beverages (SSBs) is associated with obesity, type 2 diabetes, and cardiovascular disease. SSB intake varies by state and individual sociodemographic characteristics.

**What is added by this report?**

This is the first report describing differences at the state level in daily SSB intake among adults residing in metropolitan and nonmetropolitan counties. The adjusted prevalence of daily SSB intake was significantly higher in nonmetropolitan counties (30.9%) than in metropolitan counties (24.8%) (adjusted prevalence ratio = 1.32). This same pattern was significant in 5 of 11 states with metropolitan and nonmetropolitan counties.

**What are the implications for public health practice?**

Public health strategies aiming to decrease SSB intake could focus on groups with more frequent SSB intake while taking into account differences by metropolitan status.

## Objective

Sugar-sweetened beverages (SSBs) are a leading source of added sugars in diets of US adults (1). Frequent SSB intake is associated with increased risk of obesity, type 2 diabetes, cardiovascular disease, and other health consequences (2). Although a pooled analysis of 9 states found higher prevalence of daily SSB intake among adults in nonmetropolitan counties than in metropolitan counties (3), state-level SSB intake among adults by metropolitan and nonmetropolitan counties is unknown. This analysis aimed to determine prevalence of daily SSB intake among adults residing in metropolitan and nonmetropolitan US counties by sociodemographic characteristics, weight status, and state.

## Methods

The Behavioral Risk Factor Surveillance System (BRFSS) is a state-based, random-digit–dialed, annual landline and cellular telephone survey of US adults aged 18 years or older conducted by state health departments with assistance from the Centers for Disease Control and Prevention (CDC) to monitor chronic health conditions and associated risk factors (4). It uses multistage stratified sampling to yield a representative sample of the civilian noninstitutionalized adult population in all 50 states, the District of Columbia, and 3 US territories. In 2017, 12 states and the District of Columbia (median BRFSS response rate, based on combined landline and cell phone data, 43.0% [range, 32.9%–54.0%] [5]) included an optional module with 2 questions on SSB intake: 1) “During the past 30 days, how often did you drink regular soda or pop that contains sugar? Do not include diet soda or diet pop.” and 2) “During the past 30 days, how often did you drink sugar-sweetened fruit drinks (such as Kool-Aid and lemonade), sweet tea, and sports or energy drinks (such as Gatorade and Red Bull)? Do not include 100% fruit juice, diet drinks, or artificially sweetened drinks.” Respondents reported number of times per month, week, or day; all responses were converted to daily intake, and responses for the 2 questions were summed to calculate total SSB intake. We categorized frequency of total SSB intake as none, more than 0 to fewer than 1 time per day, and 1 or more times per day. Of 80,662 adult respondents, we excluded 11,766 (14.6%; range, 10.4%–26.1%) who had missing responses to either SSB question. Our analysis included 68,896 adults who responded to both questions in the SSB module. We calculated unadjusted prevalence estimates overall, and by age, sex, race/ethnicity, education, employment status, weight status, and state. We used χ^2^ tests to determine whether SSB intake differed by covariates (significant at *P* < .05).

We used the National Center for Health Statistics Urban–Rural Classification Scheme for Counties to classify levels of urbanization of counties (6). We further dichotomized counties into metropolitan (large central metro, large fringe metro, medium metro, and small metro) and nonmetropolitan (micropolitan and noncore) (7). Delaware and the District of Columbia do not have nonmetropolitan counties; thus, we calculated SSB intake frequencies only for metropolitan counties in these areas. We determined adjusted prevalence ratios (APRs) and 95% confidence intervals (CIs) and compared SSB intake (<1 time per day and ≥1 time per day, with a reference of 0 times per day) between metropolitan and nonmetropolitan counties by using multinomial logistic regression, controlling for age, sex, and race/ethnicity within population subgroups (significant at *P* < .05). All analyses were performed by using SAS version 9.4 (SAS Institute) and accounted for sampling weights and complex survey design.

## Results

In 2017, 26.0% of respondents reported consuming SSBs 1 or more times per day; responses ranged from 17.0% in Vermont to 40.1% in Arkansas (Table 1). The unadjusted prevalence of SSB intake differed significantly by each sociodemographic characteristic and by weight status (Table 1; all *P* < .05). A higher percentage of adults in nonmetropolitan counties (30.9%) than in metropolitan counties (24.8%) reported daily SSB intake (APR = 1.32; 95% CI, 1.26–1.39; *P* < .001) (Table 2). The largest APRs for daily SSB intake between adults in nonmetropolitan counties and adults in metropolitan counties, by sociodemographic characteristic, were the following: by age, among adults aged 25 to 34 (APR = 1.45; 95% CI, 1.27–1.65); by sex, among men (APR = 1.33; 95% CI, 1.25–1.43); by race/ethnicity, among non-Hispanic others (APR = 2.01; 95% CI, 1.69–2.39); by education, among college graduates (APR = 1.51; 95% CI, 1.33–1.70); and by employment status, among employed persons (APR = 1.39; 95% CI, 1.30–1.49). At the state level, daily SSB intake in nonmetropolitan counties ranged from 17.0% in Vermont to 44.1% in Arkansas (Table 2), and in metropolitan counties SSB intake ranged from 17.1% in Vermont to 37.6% in West Virginia. Adjusted daily SSB intake was significantly higher among adults in nonmetropolitan than metropolitan counties in 5 of 11 states.

**Table 1:** Prevalence of Sugar-Sweetened Beverage (SSB)^a^ Intake Among Adults (≥18 Years), by Sociodemographic Characteristics and State, Behavioral Risk Factor Surveillance System, 12 States and the District of Columbia, 2017

Characteristic	Total, n (%)^b^	Unadjusted Prevalence, Weighted % (95% Confidence Interval)^c^		
		Sugar-Sweetened Beverage Intake		
		None	More Than 0 But <1 Time per Day	≥ 1 Time per Day
**Total sample**	68,896 (100.0)	29.2 (28.5–29.8)	44.8 (44.1–45.6)	26.0 (25.3–26.7)
**Age group, y (n = 68,896)**				
18–24	3,360 (4.9)	12.0 (10.0–14.4)	54.2 (51.2–57.1)	33.8 (31.2–36.6)
25–34	6,356 (9.2)	15.9 (14.4–17.6)	51.5 (49.3–53.7)	32.6 (30.6–34.6)
35–54	18,615 (27.0)	26.2 (25.1–27.4)	44.9 (43.6–46.3)	28.8 (27.6–30.0)
≥55	40,565 (58.9)	42.0 (41.1–43.0)	39.2 (38.3–40.2)	18.7 (18.0–19.5)
**Sex (n = 68,854)**				
Male	30,048 (43.6)	23.5 (22.5–24.4)	46.4 (45.2–47.5)	30.2 (29.2–31.2)
Female	38,806 (56.4)	34.5 (33.5–35.5)	43.4 (42.3–44.4)	22.1 (21.3–23.0)
**Race/ethnicity (n = 67,750)**				
Non-Hispanic white	49,827 (73.5)	32.2 (31.4–32.9)	43.3 (42.5–44.2)	24.5 (23.8–25.3)
Non-Hispanic black	5,869 (8.7)	19.9 (17.8–22.1)	46.4 (43.7–49.1)	33.7 (31.2–36.3)
Hispanic	4,453 (6.6)	21.4 (19.3–23.6)	48.9 (46.3–51.5)	29.7 (27.5–32.1)
Non-Hispanic other	7,601 (11.2)	25.9 (23.3–28.7)	49.7 (46.7–52.7)	24.4 (22.0–27.0)
**Education (n = 68,683)**				
<High school graduate	4,527 (6.6)	23.7 (21.6–26.0)	38.0 (35.4–40.7)	38.3 (35.8–40.9)
High school graduate	18,961 (27.6)	25.2 (24.0–26.5)	41.2 (39.7–42.6)	33.6 (32.3–35.0)
Some college	18,904 (27.5)	28.0 (26.8–29.3)	46.8 (45.4–48.2)	25.2 (24.0–26.4)
College graduate	26,291 (38.3)	36.9 (35.8–38.1)	49.4 (48.2–50.7)	13.6 (12.8–14.5)
**Employment status (n = 68,482)**				
Employed	33,481 (48.9)	25.0 (24.1–25.9)	47.7 (46.7–48.8)	27.3 (26.3–28.2)
Not employed	13,025 (19.0)	26.2 (24.7–27.8)	43.8 (42.0–45.6)	30.0 (28.5–31.6)
Retired	21,976 (32.1)	44.5 (43.1–45.8)	37.8 (36.5–39.1)	17.8 (16.8–18.8)
**Weight status^d^ (n = 64,931)**				
Underweight/normal weight	21,608 (33.3)	30.6 (29.4–31.9)	44.8 (43.3–46.2)	24.6 (23.4–25.8)
Overweight	23,097 (35.6)	29.8 (28.6–31.0)	45.4 (44.1–46.7)	24.8 (23.7–25.9)
Obesity	20,226 (31.1)	26.6 (25.5–27.8)	43.8 (42.4–45.1)	29.6 (28.4–30.8)
**State (n = 68,896)**				
Alaska	2,797 (4.1)	27.4 (25.0–30.0)	49.3 (46.2–52.5)	23.3 (20.5–26.3)
Arizona	12,651 (18.4)	29.4 (28.4–30.4)	43.9 (42.8–45.1)	26.7 (25.6–27.8)
Arkansas	4,506 (6.5)	20.7 (19.0–22.6)	39.2 (36.6–41.9)	40.1 (37.3–42.8)
Delaware	3,517 (5.1)	28.2 (26.3–30.3)	45.4 (43.1–47.8)	26.4 (24.3–28.5)
District of Columbia	3,910 (5.7)	29.3 (27.5–31.2)	45.8 (43.7–47.9)	24.9 (23.1–26.8)
Hawaii	6,723 (9.8)	30.6 (29.1–32.2)	48.9 (47.2–50.5)	20.5 (19.1–22.0)
Iowa	3,220 (4.7)	29.5 (27.8–31.3)	43.8 (41.8–45.8)	26.7 (24.9–28.6)
New York	4,979 (7.2)	34.5 (32.8–36.2)	47.7 (45.8–49.6)	17.9 (16.4–19.4)
North Carolina	3,838 (5.6)	22.2 (20.6–23.9)	40.8 (38.8–42.9)	37.0 (34.9–39.1)
Ohio	7,237 (10.5)	28.8 (27.3–30.2)	43.9 (42.2–45.7)	27.3 (25.8–29.0)
Vermont	5,692 (8.3)	38.4 (36.7–40.1)	44.6 (42.8–46.4)	17.0 (15.5–18.6)
West Virginia	5,020 (7.3)	22.3 (21.0–23.6)	39.4 (37.7–41.1)	38.3 (36.6–40.1)
Wisconsin	4,806 (7.0)	27.6 (25.9–29.2)	47.7 (45.7–49.7)	24.7 (23.0–26.6)

a Includes regular soda, sugar-sweetened fruit drinks, sweet tea, sports drinks, and energy drinks. Does not include 100% fruit juice, diet soda, diet drinks, or artificially sweetened drinks.

b Unweighted sample size and weighted percentage. Percentages might not sum to 100% because of rounding.

c The distribution of sugar-sweetened beverage consumption differed significantly by sociodemographic characteristics, weight status, and state (*P* < .05 for all by χ^2^ test).

d Based on body mass index (BMI), calculated as weight in kilograms divided by height in meters squared: underweight/normal weight (BMI <25.0 kg/m^2^), overweight (BMI 25.0 to <30.0 kg/m^2^), obesity (BMI ≥30.0 kg/m^2^).

**Table 2:** Prevalence of Sugar-Sweetened Beverage^a^ Intake ≥1 Time Per Day Among Adults (≥18 Years), by Sociodemographic Characteristics and State, Behavioral Risk Factor Surveillance System, 12 States and the District of Columbia, 2017

Characteristic	Total, n (%)^b^	Unadjusted Prevalence of Intake ≥1 Time Per Day, Weighted % (95% Confidence Interval)		Adjusted Prevalence Ratio^d ^(95% Confidence Interval)
		Metropolitan^c^	Nonmetropolitan^c^	
**Total sample**	68,896 (100.0)	24.8 (24.0–25.6)	30.9 (29.7–32.2)	1.32 (1.26–1.39)^e^
**Age group, y (n = 68,896)**				
18–24	3,360 (4.9)	32.9 (29.9–36.0)	38.4 (33.0–44.1)	1.22 (1.04–1.45)^e^
25–34	6,356 (9.2)	30.4 (28.2–32.7)	42.5 (38.0–47.1)	1.45 (1.27–1.65)^e^
35–54	18,615 (27.0)	27.0 (25.7–28.4)	36.7 (34.4–39.0)	1.39 (1.28–1.51)^e^
≥55	40,565 (58.9)	18.0 (17.1–18.9)	21.4 (20.1–22.9)	1.23 (1.13–1.33)^e^
**Sex (n = 68,854)**				
Male	30,048 (43.6)	28.6 (27.5–29.8)	36.4 (34.4–38.5)	1.33 (1.25–1.43)^e^
Female	38,806 (56.4)	21.2 (20.2–22.3)	25.8 (24.3–27.5)	1.31 (1.22–1.42)^e^
**Race/ethnicity (n = 67,750)**				
Non-Hispanic white	49,827 (73.5)	22.8 (21.9–23.7)	29.9 (28.5–31.3)	1.32 (1.25–1.41)^e^
Non-Hispanic black	5,869 (8.7)	33.5 (30.8–36.3)	36.3 (30.4–42.6)	1.12 (0.93–1.34)
Hispanic	4,453 (6.6)	29.7 (27.3–32.2)	30.3 (23.9–37.5)	1.01 (0.78–1.30)
Non-Hispanic other	7,601 (11.2)	21.3 (18.6–24.2)	41.2 (36.3–46.3)	2.01 (1.69–2.39)^e^
**Education (n = 68,683)**				
<High school graduate	4,527 (6.6)	36.9 (34.0–39.9)	43.7 (39.0–48.5)	1.12 (0.97–1.29)
High school graduate	18,961 (27.6)	32.8 (31.2–34.5)	36.0 (33.8–38.3)	1.13 (1.04–1.22)^e^
Some college	18,904 (27.5)	24.6 (23.2–26.0)	27.5 (25.4–29.6)	1.19 (1.08–1.31)^e^
College graduate	26,291 (38.3)	13.0 (12.1–13.9)	18.0 (16.2–20.0)	1.51 (1.33–1.70)^e^
**Employment status (n = 68,482)**				
Employed	33,481 (48.9)	25.7 (24.7–26.8)	33.9 (32.0–35.8)	1.39 (1.30–1.49)^e^
Not employed	13,025 (19.0)	28.6 (26.8–30.5)	35.7 (32.8–38.6)	1.25 (1.13–1.39)^e^
Retired	21,976 (32.1)	17.4 (16.2–18.7)	19.1 (17.3–21.0)	1.14 (1.01–1.28)^e^
**Weight status^f^** **(n = 64,931)**				
Underweight/normal weight	21,608 (33.3)	23.3 (22.0–24.7)	30.3 (27.8–32.9)	1.36 (1.23–1.51)^e^
Overweight	23,097 (35.6)	23.4 (22.1–24.7)	30.4 (28.2–32.7)	1.36 (1.24–1.49)^e^
Obesity	20,226 (31.1)	28.5 (27.1–30.0)	33.3 (31.2–35.5)	1.23 (1.13–1.33)^e^
**State (n = 68,896)**				
Alaska	2,797 (4.1)	22.6 (19.1–26.5)	24.6 (20.4–29.2)	1.06 (0.83–1.34)
Arizona	12,651 (18.4)	26.3 (25.2–27.4)	34.9 (30.8–39.3)	1.40 (1.23–1.59)^e^
Arkansas	4,506 (6.5)	37.0 (33.5–40.8)	44.1 (40.0–48.4)	1.22 (1.07–1.38)^e^
Delaware	3,517 (5.1)	26.4 (24.3–28.5)	—g	—g
District of Columbia	3,910 (5.7)	24.9 (23.1–26.8)	—g	—g
Hawaii	6,723 (9.8)	20.4 (18.8–22.2)	20.8 (18.4–23.4)	1.12 (0.96–1.30)
Iowa	3,220 (4.7)	26.4 (23.9–29.1)	27.1 (24.6–29.8)	1.11 (0.97–1.28)
New York	4,979 (7.2)	17.8 (16.3–19.4)	18.2 (14.4–22.8)	1.27 (1.00–1.62)
North Carolina	3,838 (5.6)	36.0 (33.5–38.6)	39.9 (36.2–43.8)	1.13 (1.01–1.27)^e^
Ohio	7,237 (10.5)	26.4 (24.5–28.3)	30.5 (27.7–33.5)	1.21 (1.07–1.37)^e^
Vermont	5,692 (8.3)	17.1 (14.3–20.3)	17.0 (15.3–18.7)	1.05 (0.86–1.28)
West Virginia	5,020 (7.3)	37.6 (35.4–39.9)	39.4 (36.7–42.2)	1.06 (0.97–1.16)
Wisconsin	4,806 (7.0)	23.3 (21.2–25.5)	28.5 (25.3–31.8)	1.32 (1.15–1.52)^e^

a Includes regular soda, sugar-sweetened fruit drinks, sweet tea, sports drinks, and energy drinks. Does not include 100% fruit juice, diet soda, diet drinks, or artificially sweetened drinks.

b Unweighted sample size and weighted percentage. Percentages might not sum to 100% because of rounding.

c Metropolitan and nonmetropolitan status based on National Center for Health Statistics Urban–Rural Classification Scheme for Counties (6,7). Metropolitan includes large central metro, large fringe metro, medium metro, and small metro categories. Nonmetropolitan includes micropolitan and noncore categories.

d Prevalence ratios were determined by using multinomial logistic regression, controlling for age, sex, and race/ethnicity. Multinomial logistic regression modeled the adjusted prevalence ratio of >0 to <1 SSB intake per day and ≥1 SSB intake per day versus a reference of 0 times per day. Only adjusted prevalence ratios for SSB intake ≥1 time per day are presented here.

e Significant ratio in the prevalence of obesity between metropolitan and nonmetropolitan areas at the *P* < .05 level based on multinomial logistic regression within levels of sociodemographic characteristics and states controlling for age, sex, and race/ethnicity.

f Based on body mass index (BMI), calculated as weight in kilograms divided by height in meters squared: underweight/normal weight (BMI <25.0 kg/m^2^), overweight (BMI 25.0 to <30.0 kg/m^2^), obesity (BMI ≥30.0 kg/m^2^).

g Data not available because no counties in Delaware or the District of Columbia were classified as nonmetropolitan.

## Discussion

In 2017, 26.0% of adults in 12 states and the District of Columbia reported consuming SSBs 1 or more times per day. Frequency of daily SSB intake was significantly higher in nonmetropolitan counties than in metropolitan counties, with approximately 1 in 3 adults in nonmetropolitan counties and 1 in 4 adults in metropolitan counties consuming SSBs 1 or more times per day. We found this significant association in 5 of 11 surveyed states that had nonmetropolitan counties.

The reported prevalence of daily SSB intake was lower than the prevalence reported in BRFSS 2016 data, in which 32.1% of US adults in 9 states reported consuming SSBs 1 or more times per day (3). This discrepancy could be attributed to geographic differences: only 5 states were included in both analyses. Differences in SSB marketing, access, availability, or pricing may exist in different communities (8,9). Consistent with previous studies, our study showed higher prevalence of daily SSB intake among younger adults, men, non-Hispanic adults, less educated adults, and unemployed adults (3,10–12). However, ours is the first report describing state-level SSB intake among US adults by metropolitan status.

Our findings are subject to limitations. First, only 12 states and the District of Columbia included the optional SSB module in 2017, and the response rate was relatively low, although the data were adjusted for some factors related to nonresponse. Therefore, our findings may not be generalizable nationwide. Second, SSB intake was measured by 2 questions that did not specify other SSB sources, such as sweetened coffees. The 2 questions measured frequency instead of volume; thus, total amount of SSB intake could not be determined. Third, our analysis did not control for education, employment, or other characteristics potentially associated with metropolitan status. Finally, self-reported data may be subject to recall or social desirability bias.

Decreasing SSB intake could reduce the burden of chronic diseases among US adults. Strategies to decrease SSB intake should consider subgroups with high intake, such as people with less education, and seek to overcome challenges in nonmetropolitan counties. Our findings could inform efforts that aim to reduce frequent SSB intake in nonmetropolitan areas.
